# Detection of SARS-CoV-2 in conjunctival secretion and tears in patients with COVID-19 in a tertiary care centre, South India. .

**DOI:** 10.12688/f1000research.123556.1

**Published:** 2022-09-16

**Authors:** Rajesh R. Nayak, Sevitha Bhat, Ajay R Kamath, Anshul Chandak, Kanishk Khare

**Affiliations:** 1 Opthalmology, Kasturba Medical College, Mangalore, Manipal Academy of Higher Education, Manipal, Mangalore, Karnataka, 575001, India; 2 Microbiology, Kasturba Medical College, Mangalore, Manipal Academy of Higher Education, Manipal, India, MANGALORE, Karnataka, 575001, India

**Keywords:** Conjunctival swab, nasopharyngeal swab, SARS-CoV-2, COVID-19, RT-PCR

## Abstract

**Aims and objectives**: Purpose of this study is to detect the presence of SAR-CoV-2 viral RNA in conjunctival secretions of COVID-19 patients and to compare the RT-PCR positivity rate for SARS-CoV-2 in conjunctival and nasopharyngeal swab.

**Materials and method**: Eighty hospitalised COVID-19 patients whose nasopharyngeal swab tested positive for SARS-CoV-2 by RT-PCR were included in the study. Conjunctival swab was collected from eyes of these patients and sent for detection of SARS-CoV-2 by RT-PCR method.

**Results**: Among the eighty patients, 51 (63.7%) were males and 29 (36.3%) were females. The mean age of the patients was 55.93 ± 16.59. Six patients had ocular manifestations. Eleven (13.75%) patients tested positive on conjunctival swab for SARS-CoV-2 viral RNA and only one of them had ocular manifestations out of the eleven.

**Conclusion**: In our study the presence of SARS-CoV-2 in conjunctival secretions of COVID-19 patients was detected and this was not dependent on presence of ocular manifestations or duration of disease. Though the conjunctival positivity is lower compared to the nasopharyngeal swab sampling, ocular surface and secretions can be a potential route of viral transmission.

## Introduction

An outbreak of pneumonia of unknown cause was first reported in late December 2019 from Wuhan, China. A new variant of coronavirus temporarily called 2019 novel coronavirus (2019-nCoV) was found to be the culprit behind it. ^1^ This non-segmented enveloped RNA virus of the *Coronaviridae* family was named ‘severe acute respiratory syndrome coronavirus 2 (SARS-CoV-2)’ as it resembled the virus causing the 2003 SARS outbreak, genetically, and the disease was termed as COVID-19 (Coronavirus disease). ^2^^,^^3^

Clinically majority of patients have symptoms of fever, dry cough, and breathlessness while the less common symptoms include headache, fatigue, confusion, and diarrhoea. Though the majority of patients are asymptomatic or have mild to moderate influenza-like illness, severe cases of COVID-19 may present with a severe lung infection, acute respiratory or renal injury, septic shock, etc. ^4^^,^^5^ Ground-glass opacity was a typical finding on the chest computed tomography which was seen in the majority of the patients. Decreased lymphocytes were another commonly seen finding on blood investigation. ^6^ Alteration in taste and smell sensations has also been reported by patients with milder symptoms. ^7^ Eye symptoms are also reported in a few patients which included watering, redness, itching, and discharge. ^8^

SARS-CoV-2 led to a global health crisis causing COVID-19. Worldwide a total of 220 million individuals have been diagnosed with the presence of the virus, and approximately 4.5 million people among them have been lost to COVID-19. In India alone, approximately 33 million individuals were found positive for SARS-CoV-2, and approximately half a million individuals have died. Though, the vaccination drive is at its height still the cases of COVID-19 are on the rise. The transmission of the coronavirus appears mainly through respiratory droplets and direct contact as the major routes for infection. Transmission through the gastrointestinal route, aerosol and eye secretions, is still to be further investigated. The possibility of transmission of the virus through ocular surfaces like the conjunctiva and cornea is still unproven. ^9^^–^^12^ This highly contagious disease has presented as a medical challenge in terms of diagnosing and management because of the wide spectrum of disease symptoms with which a patient presents. RT-PCR (Reverse Transcription Polymerase Chain Reaction) test to look for SARS-CoV-2 in swabs taken from the nasopharyngeal area is still the gold standard for diagnosing COVID-19 disease. ^13^

The ocular surface may be an inoculation site by direct touch or aerosols. From the conjunctiva, the virus may further spread to the cornea or may spread through the tears and secretions draining into the nasolacrimal duct to finally reach the nasopharynx. There have been few studies conducted worldwide demonstrating the presence of viruses in ocular secretions.

### Objectives

In our study, we aimed towards detecting SARS-CoV-2 in the conjunctival secretions and tears of COVID-19 affected individuals via reverse-transcriptase polymerase chain reaction (RT-PCR). The viral presence in the tears and ocular secretions may suggest another potential route of virus transmission. Furthermore, it will support the importance of avoiding touching of eyes and the use of protective eyewear among the doctors and even the general population at large.

## Methods

Study design and setting: It was a prospective cross-sectional study conducted in a tertiary care set up with COVID-19 care in the southern state of India between June 2021 and July 2021.

The study commenced with Institutional level ethical clearance from the Institutional Ethics Committee, Kasturba Medical College, Mangalore with the approval number IEC KMC MLR06-2021/191.

### Study participants

Patients admitted with a positive nasopharyngeal swab for SARS-CoV-2 within a duration of 1 week, and age of more than 18 years. Patients with Covid-19 like symptoms but whose RT-PCR was negative for Covid-19 were excluded. Critically ill patients who couldn’t give consent were also excluded.

### Variables and data source

Patients presenting complaints were noted along with any ocular complaints. The patient’s co-morbidities were recorded and clinical parameters on presentation like body temperature and oxygen saturation (SpO2) were also taken from the patient’s medical record. Day of nasopharyngeal swab collection was recorded and days between the nasopharyngeal swab collection and conjunctival swab collection was calculated for every patient. Written informed consent was obtained from patients before sample collection.

### Sample collection

A bedside ophthalmologist examination was done and the patient’s eyelids, adnexa, conjunctiva, and cornea were examined before collection of samples. The sample was collected under aseptic conditions by the same ophthalmologist who was donned in personal protective equipment. Samples were collected by using nylon flocked swabs by sweeping movement over the inferior fornix from the medial to the lateral direction. Sample from both eyes was collected for each patient using separate swab sticks without the use of any topical anaesthetic drops. Both swab sticks were then dipped into a single viral transport medium and transferred to the institutional microbiology lab, maintaining a proper cold chain at all times. The RTPCR was performed on the samples using the kit protocol and the results were recorded.

### Statistical analysis

All the data was entered into an excel sheet and analyzed using IBM SPSS version 25. The continuous and categorical variables have been represented as mean ± standard deviation and frequency percentages respectively. The association between conjunctival swab positivity and variables like the severity of COVID, presence of symptoms such as fever, breathlessness, cough, fatigue, myalgia, diarrhoea, and ocular symptoms were analyzed using the chi-square test. Correlation between the duration of days after which conjunctival swab is tested positive from the day of nasopharyngeal swab positivity, and oxygen saturation was done using Pearson’s correlation and p<0.05 was considered significant.

## Results

A total of 80 patients who were tested positive for SARS-CoV-2 on nasopharyngeal swabs were included in our study. Among them 51(63.7%) were males and 29(36.3%) were females. The mean age of the patients was 55.93 years with a standard deviation of 16.59 years. The average time interval between conjunctival swab collection and nasopharyngeal swab collections was similar in overall patients when compared to patients with conjunctival swab positivity with an average interval of 3.96±2.25 days and 3.45±2.73 days respectively. The mean of baseline parameters (like body temperature, respiratory rate, and oxygen saturation) of patients with conjunctival swab positivity was also similar to that of overall study patients ( Table 1).

**Table 1.** Descriptive statistics for continuous variables for the whole sample and conjunctival swab positive patients.

Variable	Study sample (n=80)		conjunctival swab positive patients (n=11)	
	Mean	Std deviation	Mean	Std deviation
Age	55.93	16.59	56.36	18.41
Duration from Nasopharyngeal swab	3.96	2.25	3.45	2.73
Duration of hospital stay till conjunctival swab	4.23	2.27	3.09	2.07
Temp	98.42	1.12	97.90	.70
RR	19.20	3.55	18.18	3.15
SpO _2_	95.09	4.41	94.09	4.08

The presence of COVID-19 related symptoms did not show any variation in distribution among conjunctival swab-positive patients as compared to the whole study sample. Fever was the most common symptom among these patients seen in 70% of patients followed by cough (60%), breathlessness (52.5%), fatigue (36.3%), myalgia (18.8%), diarrhoea (8.8%), and ocular symptoms (7.5%) ( Table 2). The association between Conjunctival swab positivity and the severity of COVID-19 infection is shown in Table 3.

**Table 2.** Descriptive statistics for categorical variables for the whole sample and conjunctival swab positive patients.

Variable	Categories	Study sample (n=80)		conjunctival swab positive patients (n=11)	
		Frequency	Percent	Frequency	Percent
**Sex**	Male	51	63.7	7	63.6
	Female	29	36.3	4	36.4
**Conjunctival swab positivity**	Negative	69	86.3	0	0
	Positive	11	13.8	11	100
**Category**	Mild	40	50.0	5	45.5
	Moderate	27	33.8	4	36.4
	Severe	13	16.3	2	18.2
**Fever**	Absent	24	30.0	4	36.4
	Present	56	70.0	7	63.6
**Breathlessness**	Absent	38	47.5	5	45.5
	Present	42	52.5	6	54.5
**Cough**	Absent	32	40.0	4	36.4
	Present	48	60.0	7	63.6
**Fatigue**	Absent	51	63.7	7	63.6
	Present	29	36.3	4	36.4
**Myalgia**	Absent	63	78.8	7	63.6
	Present	15	18.8	4	36.4
**Diarrhoea**	Absent	73	91.3	11	100.0
	Present	7	8.8	0	0
**Ocular symptoms**	Absent	74	92.5	10	90.9
	Present	6	7.5	1	9.1
**DM**	Absent	42	52.5	7	63.6
	Present	38	47.5	4	36.4
**HTN**	Absent	48	60.0	7	63.6
	Present	32	40.0	4	36.4
**COPD**	Absent	69	86.3	10	90.9
	Present	9	11.3	0	0
**IHD**	Absent	72	90.0	9	81.8
	Present	5	6.3	0	0
**Xray or CT findings**	Absent	60	75.0	7	63.6
	Present	20	25.0	4	36.4

**Table 3.** Conjunctival swab positivity * Category.

Conjunctival swab positivity	Count/%	Category			Chi square (p value)
		Mild	Moderate	Severe	
Absent	Count	35	23	11	.108 (0.94)
	%	50.7%	33.3%	15.9%	
Present	Count	5	4	2	
	%	45.5%	36.4%	18.2%	

Inference: Conjunctival swab positivity was not associated with severity of COVID.

A total of 11 patients showed conjunctival swab positivity for SARS-CoV-2 by RT-PCR test. Among them, only one patient had ocular symptoms as compared to five patients complaining of ocular symptoms in whom conjunctival swab showed a negative result for the presence of the concerned virus. There was no correlation found between conjunctival swab positivity and the presence of ocular symptoms by using the chi-square test ( Table 4).

**Table 4.** Conjunctival swab positivity * Ocular symptoms.

Conjunctival swab positivity	Count/%	Ocular symptoms		Chi square (p value)
		Absent	Present	
Absent	Count	64	5	0.04 (.82)
	%	92.8%	7.2%	
Present	Count	10	1	
	%	90.9%	9.1%	

Inference: Conjunctival swab positivity was not associated with the presentation of ocular symptoms.

Conjunctival swab positivity was not correlated to the time interval between conjunctival swab collection from that of nasopharyngeal swab collection as shown by Spearman's correlation. This shows conjunctival swab positivity is not affected by disease duration among COVID-19 patients ( Table 5).

**Table 5.** Correlation between conjunctival positivity and duration from nasal swab.

Conjunctival positivity	Mean duration from nasal swab	Std deviation	Spearmans correlation coefficient (rho)	P value
Absent	4.04	2.179	-.091	.422
Present	3.45	2.734		

Inference: Conjunctival swab positivity was not related to duration from nasal swab.

## Discussion

One of the earliest studies conducted in China by Zang *et al* demonstrated SARS-CoV-2 on the ocular surface of 102 patients, among which only two patients tested positive for the RNA virus. Ping Wu *et al* also conducted a similar study and found two (5.2%) out of 28 patients who were positive for the virus on nasopharyngeal swabs. Similar studies were also performed worldwide to investigate ocular surface and secretions as the probable transmission source of SARS-CoV-2. K Kumar *et al* were among the first to study the conjunctival presence of SARS-CoV2 in the Indian population and found one (2.23%) conjunctival swab positive out of 45 samples studied. ^3^

In our study, out of 80 nasopharyngeal swab-positive patients, 11(13.75%) patients have detected the concerned virus in their conjunctival secretions also. Our study showed a higher conjunctival swab positivity rate as compared to a few of the previously conducted studies.

Similar results were found by H Kaya *et al*, where they studied the prevalence of the virus among 32 COVID-19 patients with a positivity rate of 16% (five out of 32) was found. None of them showed conjunctivitis. Interestingly, at the time of conjunctival swab collection, a repeat nasopharyngeal swab was also sent. Out of five conjunctival swab-positive patients, two were tested negative on the nasopharyngeal swab test. ^14^ This may indicate the possibility of viral transmission through ocular surface and secretions, even after nasopharyngeal swab results are negative.

In another study conducted among the Indian population from the Northern parts, found a higher prevalence of conjunctival positivity of 24% (18 patients) in a study population of 75 patients with moderate to severe COVID-19 disease without any ocular symptoms. ^11^ Claudio Azzolini *et al,* also concluded a higher prevalence in a cohort of 91 patients, with 52 patients (57.1%) having conjunctival swab positivity for SARS-CoV-2. ^15^

The patients included in our study had a mean age of 55.93 years which was comparable to the conjunctival swab positive group of patients, 56.36 years. The systemic symptoms in the majority of patients were fever (70%), cough (60%), and breathlessness (52.5%). The mean SpO _2_ of the patients in the conjunctival swab positive group was slightly lower (94%) as compared to the patients who were negative for the same (95%).

Various studies have been conducted to compare the ocular manifestations, mainly conjunctivitis, and compare it with the positivity rate of detection of the virus in their conjunctival secretions. Noemi Güemes-Villahoz *et al*, in Spain, studied 36 COVID-19 patients, with 18 patients each in conjunctivitis and non-conjunctivitis group, and found a similar prevalence of 5.5% (one patient) of conjunctival positivity in each group. ^16^

Mahmoud H *et al* also studied 28 covid patients and found a higher conjunctival positivity prevalence rate of 28.57% (eight out of 28). Among ten patients with ocular symptoms, three tested positive on conjunctival swab while in the remaining 18 patients without ocular manifestations, five tested positive on the conjunctival swab for SARS-CoV-2. ^17^ Therefore, the authors suggested the presence of the COVID-19 virus on the ocular surface or secretions is not affected by the presence of conjunctivitis.

In our study, a total of six patients had ocular manifestations. The main symptoms were redness and watery discharge. SARS-CoV-2 was detected in the conjunctival secretions in one patient with conjunctivitis. Other five patients who had ocular symptoms tested negative for the RNA virus on conjunctival swab sampling. Ten patients among the conjunctival positive group did not have any ocular manifestations.

Prempal Kaur *et al* in a study conducted in northern India demonstrated RNA virus in conjunctival secretions of COVID 19 patients with or without ocular manifestations. The study included 60 COVID-19 patients each in two groups- one with ocular symptoms and the other without ocular symptoms (control). A similar positivity rate was found in both cases (18.33%) and control (16.66%) groups. ^18^

In our study, we also evaluated the effect of duration between the nasopharyngeal swab to the conjunctival swab collection on the viral persistence in conjunctival secretions. The mean duration in our total sample of 80 patients was 3.96 days with a standard deviation of 2.25 days, while in the conjunctival swab positive patients it was 3.45 days (2.73±), with minimum and maximum duration being one and seven days respectively. This shows that detection of virus in conjunctival secretions may vary, and is not dependent on the duration. Thus, the chance of transmission of the RNA virus through the ocular surface is possible even after seven days of presentation, when most of the systemic symptoms of COVID-19 subside.

To study the ocular route of transmission of the virus, Wei Deng *et al* conducted an animal study on rhesus monkeys and found SARS-CoV-2 inoculation on the ocular surface can cause mild Covid-19 in these monkeys suggesting transmission of the virus through the ocular route is a possibility. ^19^

The importance of protective eyewear can hence be ascertained and should be strictly practiced by doctors especially the ophthalmologist.

Our study had the limitation of a small sample size. A multi-centric and a larger cohort may be needed to further validate the ocular route of transmission of the virus. Another limitation was that the conjunctival swab was collected only once. Our study showed that the virus was detected from the conjunctival secretions as early as day one to as late as day seven from nasopharyngeal swab positivity. Therefore, results may vary with multiple sampling done over a period of time.

In conclusion, our study demonstrated SARS-CoV-2 in conjunctival secretions of COVID-19 patients and is not dependent on the presence of ocular manifestations or duration of disease. Though the conjunctival positivity is lower compared to the nasopharyngeal swab sampling, ocular surface and secretions can be a potential route of viral transmission.

## Author contributions

1. Dr Rajesh Nayak: Conceptualization, Data Curation, Formal Analysis, Funding Acquisition, Investigation,Methodology, Project Administration, Resources, Software Supervision, Validation, Visualization, Writing – Review & Editing
2. Dr Sevitha Bhat - Data Curation, Formal Analysis, Investigation, Methodology, Project Administration, Supervision, Validation, Visualization, Writing – Review & Editing
3. Dr Ajay R Kamath: Conceptualization, Funding Acquisition, Project Administration, Resources, Supervision, Validation, Visualization, Writing – Review & Editing
4. Dr Anshul Chandak: Data Curation, Formal Analysis, Investigation, Methodology, Software, Validation, Visualization, Writing – Original Draft Preparation
5. Dr Kanishk Khare: Data Curation, Formal Analysis, Investigation, Methodology, Software, Validation, Visualization, Writing – Original Draft Preparation

## Data Availability

**Dryad.** Detection of SARS-CoV-2 in conjunctival secretion and tears in patients with COVID-19 in a tertiary care centre, South India. DOI: https://doi.org/10.5061/dryad.rn8pk0pdp ^20^
