# Supplementary material for: Detection of SARS-CoV-2 in conjunctival secretion and tears in patients with COVID-19 in a tertiary care centre, South India.
Source: F1000Res. 2023 Jan 5;11:1059. Originally published 2022 Sep 16. [Version 2] doi: 10.12688/f1000research.123556.2 (PMC10066052; doi:10.12688/f1000research.123556.2)
Supplement: Peer review history

# Peer review: Reviewer response for version 2

**DOI:** 10.5256/f1000research.142625.r162563

**Published:** 2023-03-31

**Authors:** Iddya Karunasagar

**Affiliations:** 1 Technology Enabling Center, Nitte University, Deralakatte, Mangalore, India

The authors have tested the presence of SARS-CoV-2 by RT-PCR in conjunctival swabs of patients who were also tested with nasopharyngeal swabs. Data on those who tested positive and negative for conjunctival swabs were compared. Only 11/80 patients tested positive in conjunctival swabs and only one of them had ocular manifestations. This study suggests that ocular secretions can be a potential source of viral transmission.

 The study has been carried out using well-established methodology and the data has clinical significance. The results have been discussed well in the context of present status of knowledge in the field.

**Strengths of the study:** The study shows that SARS CoV2 could be present in the ocular secretions of individuals without ocular manifestations. Therefore ocular secretions should be considered a source of virus. This warrants adopting measures like handwashing and sanitising hands. This information is very important in considering pathways of transmission of SARS CoV2. The study has been performed using robust methodology.

**Weakness:** The sample size is small and this is a single center study. The weakness does not in any way minimise the significance of the finding, since even in the small sample size, a significant portion of samples were positive for SARS CoV2 virus by RT PCR in ocular secretions.

Is the work clearly and accurately presented and does it cite the current literature?

Yes

If applicable, is the statistical analysis and its interpretation appropriate?

Yes

Are all the source data underlying the results available to ensure full reproducibility?

Yes

Is the study design appropriate and is the work technically sound?

Yes

Are the conclusions drawn adequately supported by the results?

Yes

Are sufficient details of methods and analysis provided to allow replication by others?

Yes

Reviewer Expertise:

Infectious diseases

I confirm that I have read this submission and believe that I have an appropriate level of expertise to confirm that it is of an acceptable scientific standard.

# Peer review: Reviewer response for version 1

**DOI:** 10.5256/f1000research.135672.r150606

**Published:** 2022-09-28

**Authors:** Anugraha Mathew

**Affiliations:** 1 Department of Plant and Microbial Biology, University of Zurich, Zurich, Switzerland

The manuscript by Nayak *et al* *.* is original, reads well, and is of significant interest to clinicians, patients and policymakers. The authors describe the prevalence of coronavirus in the conjunctival secretions, regardless of specific ocular manifestations. Although SARS-CoV-2 conjunctivitis is rare in relation to the magnitude and prevalence of other manifestations and complications caused by the virus, the ophthalmic route of disease transmission is possible and hence this study is relevant and adds to the current knowledge of COVID-19 testing and prevention measures.

**Major comments**

- Since the objective of the study includes a comparison of the RT-PCR positivity rate for SARS-CoV-2 in conjunctival and nasopharyngeal swabs, it would be ideal to include the Ct values obtained with RT-PCR from both the samples. These values would also be a good indicator of the viral load in both samples and provide additional information to the current literature on the topic.
- The authors conclude that ocular surface and secretions can be a potential route of viral transmission. Since ACE2 receptors serve as the entry point of the viral particle, a comment or discussion on the presence of these receptors on the ocular surface would be an additional point to support this hypothesis.

**Minor comments**

**Introduction**

- **Line 1, 2nd paragraph:** " *Clinically majority of patients have symptoms of fever, dry cough, and breathlessness while the less common symptoms include headache, fatigue, confusion, and diarrhoea.*" Maybe modified as "Most patients have symptoms such as fever, dry cough, and breathlessness while the less common symptoms include headache, fatigue, confusion, and diarrhoea."
- **Line 4, 3rd paragraph: **" *The transmission of the coronavirus appears mainly through respiratory droplets and direct contact as the major routes for infection.*" Maybe modified as "According to current evidence, COVID-19 virus is primarily transmitted between people through respiratory droplets and contact routes."
- **Line 5, 3rd paragraph: **" *Transmission through the gastrointestinal route, aerosol and eye secretions, is still to be further investigated. The possibility of transmission of the virus through ocular surfaces like the conjunctiva and cornea is still unproven.*" Maybe modified as "The possibility of transmission through the gastrointestinal route, aerosol and eye secretions still needs further investigation. Likewise, the transmission of the virus through ocular surfaces like the conjunctiva and cornea is still unproven."
- **Line 7, 3rd paragraph: **" *This highly contagious disease has presented as a medical challenge...*" Maybe modified as "This highly contagious disease represents a medical challenge..."

Is the work clearly and accurately presented and does it cite the current literature?

Yes

If applicable, is the statistical analysis and its interpretation appropriate?

Yes

Are all the source data underlying the results available to ensure full reproducibility?

Partly

Is the study design appropriate and is the work technically sound?

Yes

Are the conclusions drawn adequately supported by the results?

Yes

Are sufficient details of methods and analysis provided to allow replication by others?

Yes

Reviewer Expertise:

Microbiology, Molecular Biology

I confirm that I have read this submission and believe that I have an appropriate level of expertise to confirm that it is of an acceptable scientific standard.

# Response

**Published:** 2022-09-28

**Authors:** SEVITHA BHAT

**Affiliations:** Kasturba Medical College ,Mangalore, India

**Reply to Major comments **

1. 11 of the patients with conjunctival swab positivity had the Ct values for the E and RdRp genes in the range of 25-30. The Ct values of the same patients in the nasopharyngeal swabs were in the range of 15-20. Though Ct value is not an estimate of the viral load, the values were more in the conjunctival swabs compared to the nasopharyngeal swabs.
2. Previous studies have reported the presence of ACE2 in the conjunctiva, limbus, and cornea, reemphasizing the fact that conjunctiva is susceptible to SARS-CoV-2 infection. ^20^
  Reference 20 - Zhou L, Xu Z, Castiglione GM, Soiberman US, Eberhart CG, Duh EJ. ACE2 and TMPRSS2 are expressed on the human ocular surface, suggesting susceptibility to SARS-CoV-2 infection. *Ocul Surf*. 2020 Oct;18(4):537-544.

**Reply to Minor comments **

1. **Line 1, 2nd paragraph: please modify the existing sentence as **"Most patients have symptoms such as fever, dry cough, and breathlessness while the less common symptoms include headache, fatigue, confusion, and diarrhoea."
2. **Line 4, 3rd paragraph: please modify the existing sentence as **"According to current evidence, COVID-19 virus is primarily transmitted between people through respiratory droplets and contact routes."
3. **Line 5, 3rd paragraph: please modify the existing sentence as **"The possibility of transmission through the gastrointestinal route, aerosol and eye secretions still needs further investigation. Likewise, the transmission of the virus through ocular surfaces like the conjunctiva and cornea is still unproven."
4. **Line 7, 3rd paragraph: please modify the existing sentence as **"This highly contagious disease represents a medical challenge"
